# Effectiveness of a standardized electronic admission order set for acute exacerbation of chronic obstructive pulmonary disease

**DOI:** 10.1186/s12890-018-0657-x

**Published:** 2018-05-30

**Authors:** Sachin R. Pendharkar, Maria B. Ospina, Danielle A. Southern, Naushad Hirani, Jim Graham, Peter Faris, Mohit Bhutani, Richard Leigh, Christopher H. Mody, Michael K. Stickland

**Affiliations:** 1 Department of Medicine, Cumming School of Medicine, University of Calgary, Calgary, AB Canada; 2 Department of Community Health Sciences, Cumming School of Medicine, University of Calgary, Calgary, AB Canada; 3 O’Brien Institute for Public Health, Cumming School of Medicine, University of Calgary, Calgary, AB Canada; 4 Respiratory Health Strategic Clinical Network, Alberta Health Services, Edmonton, AB Canada; 5 W21C Research and Innovation Centre, Cumming School of Medicine, University of Calgary, Calgary, AB Canada; 6 Research Priorities and Implementation, Alberta Health Services, Calgary, AB Canada; 7 Division of Pulmonary Medicine, Department of Medicine, Faculty of Medicine and Dentistry, University of Alberta, Edmonton, AB Canada; 8 University of Calgary, TRW Building, Rm 3E23, 3280 Hospital Drive NW, Calgary, AB T2N 4Z6 Canada

**Keywords:** Length of stay, Clinical decision support, Chronic obstructive pulmonary disease, Quality improvement

## Abstract

**Background:**

Variation in hospital management of patients with acute exacerbation of chronic obstructive pulmonary disease (AECOPD) may prolong length of stay, increasing the risk of hospital-acquired complications and worsening quality of life. We sought to determine whether an evidence-based computerized AECOPD admission order set could improve quality and reduce length of stay.

**Methods:**

The order set was designed by a provincial COPD working group and implemented voluntarily among three physician groups in a Canadian tertiary-care teaching hospital. The primary outcome was length of stay for patients admitted during order set implementation period, compared to the previous 12 months. Secondary outcomes included length of stay of patients admitted with and without order set after implementation, all-cause readmissions, and emergency department visits.

**Results:**

There were 556 admissions prior to and 857 admissions after order set implementation, for which the order set was used in 47%. There was no difference in overall length of stay after implementation (median 6.37 days (95% confidence interval 5.94, 6.81) pre-implementation vs. 6.02 days (95% confidence interval 5.59, 6.46) post-implementation, *p* = 0.26). In the post-implementation period, order set use was associated with a 1.15-day reduction in length of stay (95% confidence interval − 0.5, − 1.81, *p* = 0.001) compared to patients admitted without the order set. There was no difference in readmissions.

**Conclusions:**

Use of a computerized guidelines-based admission order set for COPD exacerbations reduced hospital length of stay without increasing readmissions. Interventions to increase order set use could lead to greater improvements in length of stay and quality of care.

**Electronic supplementary material:**

The online version of this article (10.1186/s12890-018-0657-x) contains supplementary material, which is available to authorized users.

## Background

Chronic obstructive pulmonary disease (COPD) is a common and progressive lung disease that is characterized by shortness of breath, activity limitation, and a predisposition to exacerbations. Acute exacerbations of COPD (AECOPD) adversely affect quality of life, [1] increase the risk of disease progression, [2] and reduce survival. [3] Hospitalizations for AECOPD cost approximately USD $3.8 billion [4] and account for 51% of overall expenditures for COPD. [5] Prolonged hospital length of stay (LOS) also have a negative impact on patient function and quality of life. [6]

Evidence-based management guidelines for AECOPD have been developed, [7, 8] and include recommendations regarding pharmacotherapy and post-exacerbation care. Despite these guidelines, hospital care of patients with AECOPD remains highly variable. [9] This variation may contribute to prolonged LOS [10] that, in turn, increases the risk of hospital-acquired complications and adversely impacts quality of life. [6, 11]

Order sets are grouped medical orders intended to standardize evidence-based best practice. Computerized Physician Order Entry (CPOE) systems may improve workflow, promote appropriate testing and treatment, reduce errors and improve guideline adherence, [12–17] particularly when integrated into general order sets. [18] Standardized admission order sets have been used in other diseases with variable success at reducing hospital LOS. [14, 15]

Two observational studies have demonstrated that order sets likely improve the quality of hospital care for patients with AECOPD and reduce LOS. [13, 16] However, these studies used pre-post designs that could be influenced by secular trends in AECOPD management, and the studies did not account for the differential effects of the order set among physician groups.

The objective of this study was to determine whether the implementation of an evidence-based computerized admission order set would improve the quality of inpatient AECOPD care. A stepped wedge design was used to account for differential effects among physician groups and to minimize confounding related to the timing of order set implementation. Our hypothesis was that the implementation of a standardized order set would reduce hospital LOS of patients admitted for AECOPD without increasing emergency department (ED) or hospital readmissions. Preliminary study results have previously been reported in abstract form. [19]

## Methods

### Study design

This study is an analysis of administrative health data for a quality improvement project in which an electronic standardized admission order set for patients with AECOPD was implemented at a large, tertiary-care teaching hospital in Calgary, Alberta between March 1, 2013 and March 31, 2015. Since this was a quality improvement project, the University of Calgary Conjoint Health Research Ethics Board waived the requirement for formal ethics approval.

### Study population

Patients were included if they were: older than 45 years of age; admitted to hospital between March 1, 2013 to March 31, 2015 with an International Classification of Diseases, Tenth Revision (ICD-10-CA) code indicative of AECOPD (J42 [unspecified chronic bronchitis], J43 [emphysema], or J44 [other chronic obstructive pulmonary disease]) in the primary diagnosis field of the hospital discharge abstract database; and admitted to the pulmonary, general internal medicine or hospitalist clinical services. Patients were excluded if they were admitted to the intensive care unit or any other clinical service. Historical controls from the 12 months prior to order set implementation in each group of ordering physicians were identified using similar criteria. Additional details on the methods are provided in an additional file (see Additional file 1).

### Order set development

The AECOPD order set was based on published COPD guidelines, [7] and developed by a provincial COPD working group comprised of physicians, nurses, and respiratory therapists from a variety of clinical settings, in a series of face-to-face and teleconference meetings.

The order set contained recommended testing, medication (including suggested dosing and mode of delivery), consultations, and a priori discharge planning interventions specific to patients with AECOPD. Some interventions were pre-selected to encourage use (e.g., physiotherapy referral). The order set was built into the hospital’s existing CPOE system, Sunrise Clinical Manager (Allscripts Solutions, Chicago IL). Screenshots are provided in an additional file (see Additional file 2).

### Implementation

The order set was implemented using a stepped wedge design [20] among the three physician groups who admit patients with AECOPD: respirologists, general internists, and family physician hospitalists. It was implemented sequentially within physician groups with each group acting as its own control. Study outcome data were collected at baseline and at each implementation ‘step’.

Implementation among respirologists, general internists and hospitalists occurred in March, May and August 2013, respectively. Prior to each implementation step, the research team met with physicians and allied health staff to introduce the order set. Order set use by each individual physician was voluntary. Monthly statistics on order set use were posted in clinical areas.

### Analysis

Patient demographic, comorbidity and hospitalization data were obtained from provincial administrative data and linked to order set usage data from the CPOE system using the patient’s provincial health number. [21]

The primary outcome was hospital LOS for patients admitted during the implementation period compared to those admitted during the previous 12 months (pre-post implementation analysis). Secondary outcomes included: hospital LOS of patients admitted with and without the order set after implementation (post-implementation analysis); all-cause readmissions at 7, 30 and 90 days after discharge; ED visits at 7 and 30 days; and in-hospital mortality.

Unadjusted and adjusted median regression models were constructed to assess the impact of the order set on LOS. [22–24] Covariates in adjusted models included age, sex, and five clinically relevant comorbidities (heart failure, dementia, liver disease, renal disease, and diabetes) that were strongly associated with the Charlson Comorbidity Index (Somers’ D = 0.94). [25] Logistic regression was used to adjust 30-day readmission odds ratios for age, sex, comorbidity and admitting physician specialty.

All analyses were performed using SAS version 9.3 (Cary, NC) or R version 3.2.3; [26] *p* < 0.05 was considered statistically significant.

## Results

**Fig. 1:**
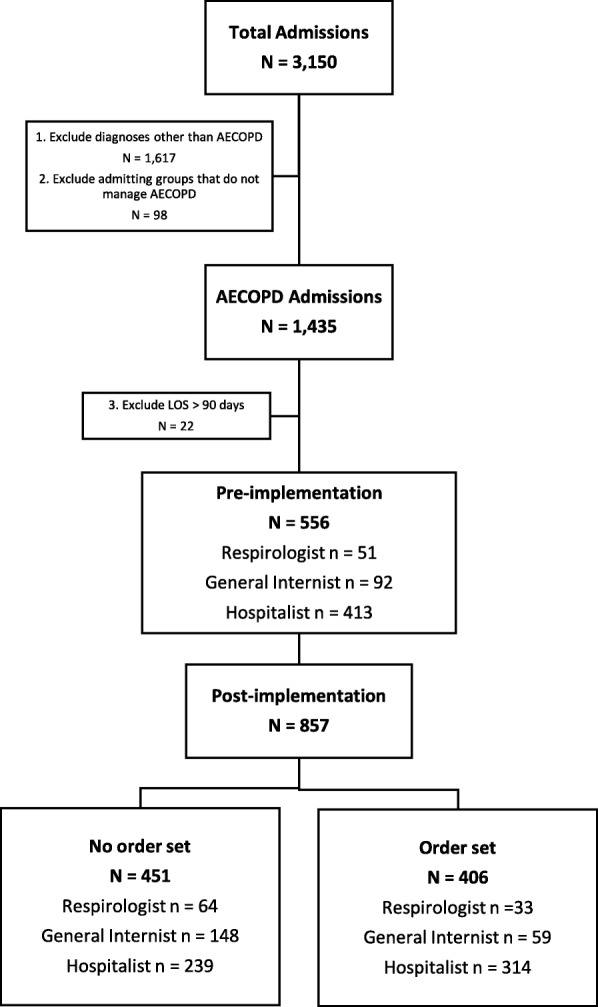
Patient flow diagram. AECOPD – acute exacerbation of chronic obstructive pulmonary disease; LOS – length of stay

Of 1435 AECOPD admissions to one of the three physician groups during the study period, 1413 with a LOS less than 90 days were included in the analysis (Fig. 1). There were 857 admissions after order set implementation, of which 406 patients (47%) were admitted using the order set.

Baseline characteristics of study participants are presented in Tables 1 and 2 for the pre-post and post-implementation analyses, respectively. The hospitalist service admitted most patients with AECOPD, but admitted fewer in the post-implementation period compared to the pre-implementation period (64.5% vs. 74.3%). Patients with co-existing heart failure and diabetes were more commonly admitted under general internists. Over 95% of patients were discharged home.

**Table 1:** Baseline characteristics for pre-post implementation analysis

Characteristics	Total	Pre-implementation	Post-implementation	*p*-value
Number of patients	1413	556	857	
Mean age, years (SD)	70 (12)	70 (12)	70 (12)	0.747
Age group, *n* (%)				
< 55	129 (9.1)	59 (10.6)	70 (8.2)	0.250
55–64	323 (22.9)	128 (23.0)	195 (22.8)	
65–74	428 (30.3)	157 (28.2)	271 (31.6)	
75–84	360 (25.5)	136 (24.5)	224 (26.1)	
85+	173 (12.2)	76 (13.7)	97 (11.3)	
Male sex, *n* (%)	727 (51.5)	279 (50.2)	448 (52.3)	0.441
Comorbidity, *n* (%)				
Heart failure	190 (13.5)	71 (12.8)	119 (13.9)	0.548
Dementia	45 (3.2)	24 (4.3)	21 (2.5)	0.051
Diabetes	318 (22.5)	131 (23.6)	187 (21.8)	0.444
Renal disease	36 (2.6)	13 (2.3)	23 (2.7)	0.687
Liver disease	13 (0.9)	6 (1.1)	7 (0.8)	0.614
Admitting specialty, *n* (%)				
Respirologist	148 (10.5)	51 (9.2)	97 (11.3)	0.0005
General internist	299 (21.2)	92 (16.6)	207 (24.2)	
Hospitalist	966 (68.4)	413 (74.3)	553 (64.5)	

*SD* = standard deviation

**Table 2:** Baseline characteristics for post-implementation analysis

Characteristics	Respirologist		General internist		Hospitalist	
	No order set (*n* = 64)	Order set (*n* = 33)	No order set (*n* = 148)	Order set (*n* = 59)	No order set (*n* = 239)	Order set (*n* = 314)
Mean age, years (SD)	63 (11)	64 (10)	69 (11)	71 (12)	72 (12)	71 (10)
Age group, *n* (%)						
< 55	8 (13)	6 (18)	12 (8)	8 (14)	15 (6)	21 (7)
55–64	27 (42)	10 (30)	37 (25)	14 (24)	39 (16)	68 (22)
65–74	20 (31)	9 (27)	50 (34)	14 (24)	72 (30)	106 (34)
75–84	9 (14)	8 (24)	36 (24)	18 (31)	65 (27)	88 (28)
85+	0	0	13 (9)	5 (9)	48 (20)	31 (10)
Male sex, *n* (%)	34 (53)	12 (36)	75 (51)	29 (49)	134 (56)	159 (51)
Comorbidity, *n* (%)						
Heart failure	4 (6)	2 (6)	39 (26)	11 (19)	37 (15)	26 (8)
Dementia	0	1 (3)	4 (3)	0	11 (5)	5 (2)
Diabetes	9 (14)	8 (24)	46 (31)	16 (27)	45 (19)	63 (20)
Renal disease	2 (3)	0	4 (3)	2 (3)	7 (3)	8 (3)
Liver disease	0	0	1 (1)	0	2 (1)	2 (1)

*SD* = standard deviation

### Order set uptake

In the post-implementation period, 57% of patients admitted to the hospitalist service were admitted using the order set, compared to 30% of patients admitted by general internists or respirologists. Time series analysis revealed that order set use increased gradually after implementation, mostly by general internists and hospitalists (Additional file 3: Figure S1).

### Hospital LOS

**Fig. 2:**
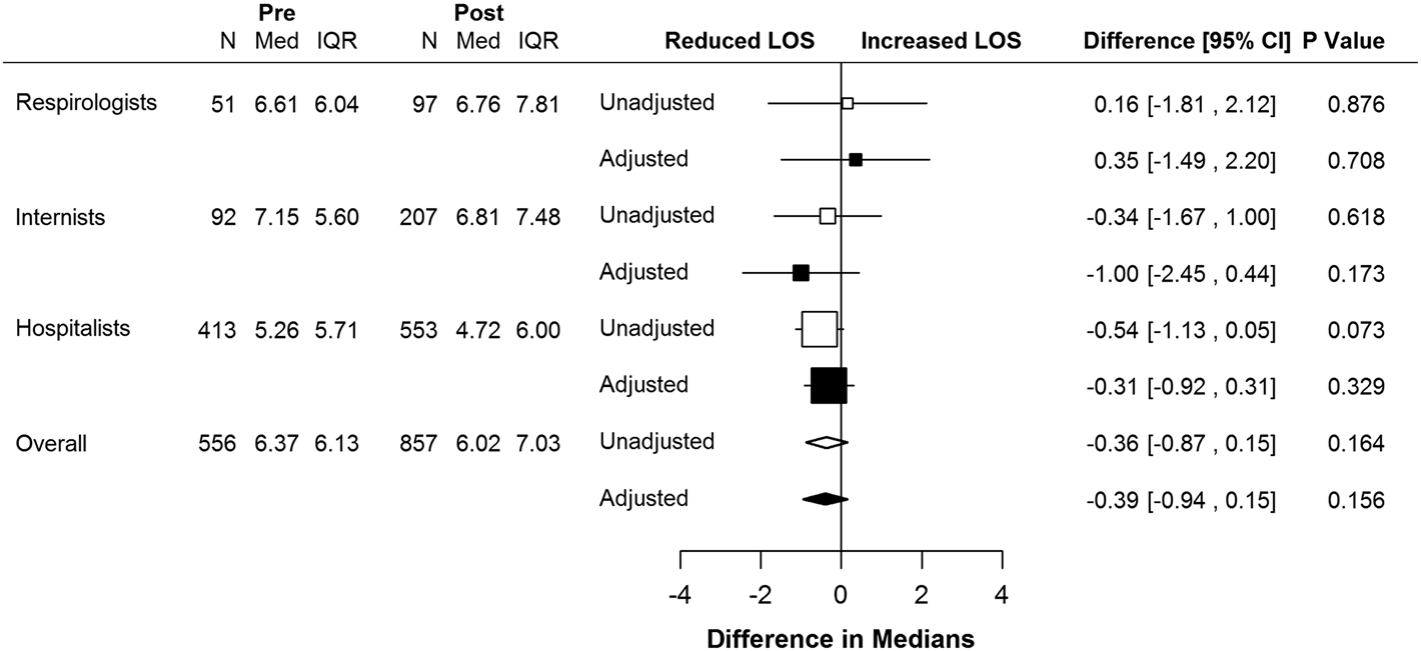
Forest plot of implementation effects (pre-post implementation analysis). Adjusted model included age, sex, and five clinically relevant comorbidities selected from the Charlson Comorbidity Index (heart failure, dementia, mild or severe liver disease, renal disease, and diabetes. Pre – pre-implementation; Post – post-implementation; N – number of patients; Med – median length of stay; IQR – interquartile range; LOS – length of stay; CI – confidence interval

Figure 2 shows the unadjusted and adjusted differences in median LOS for patients treated in the pre- and post-implementation periods. Median LOS was 6.37 days (95% confidence interval [CI] 5.94, 6.81; *n* = 556) for patients admitted before implementation and 6.02 days (95% CI 5.59, 6.46; *n* = 857) for patients admitted after implementation (*p* = 0.26).

**Fig. 3:**
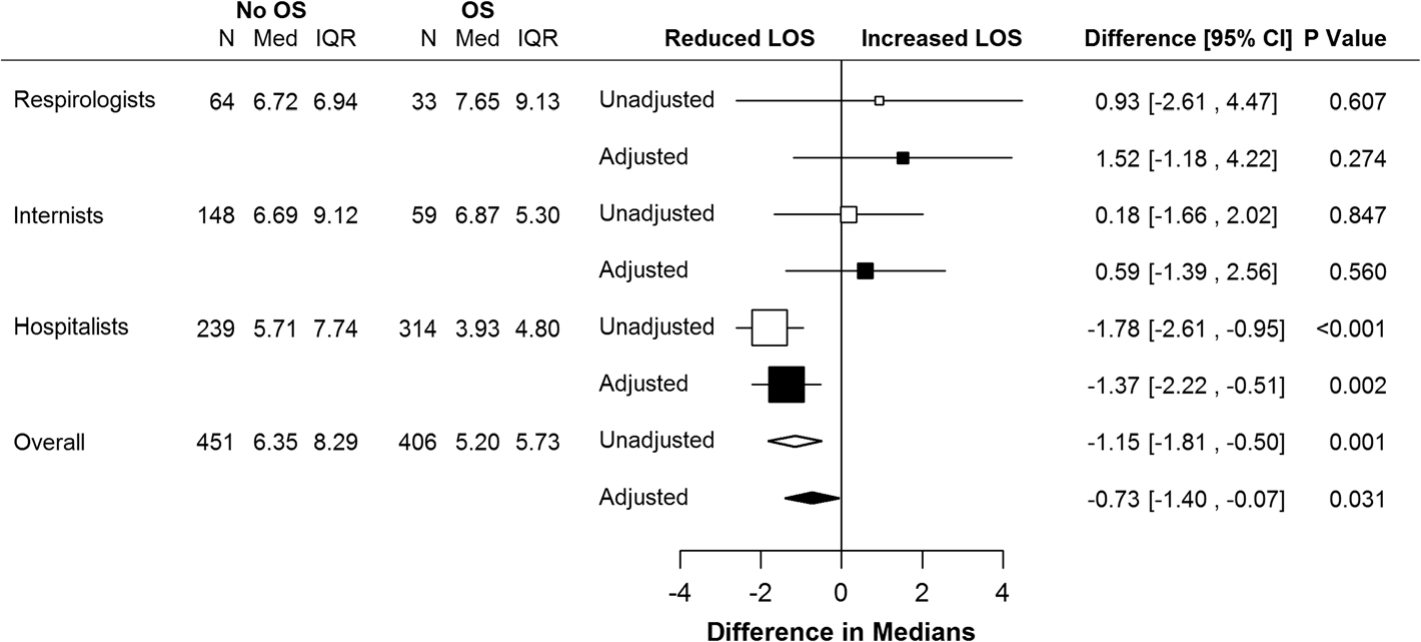
Forest plot of the effects of the order set (post-implementation analysis). Adjusted model included age, sex, and five clinically relevant comorbidities selected from the Charlson Comorbidity Index (heart failure, dementia, mild or severe liver disease, renal disease, and diabetes. OS – order set; N – number of patients; Med – median length of stay; IQR – interquartile range; LOS – length of stay; CI – confidence interval; OS – order set

Unadjusted and adjusted comparisons of median LOS in the post-implementation analysis are presented in Fig. 3. Order set use was associated with a 1.15-day (95% CI -0.50, − 1.81) shorter median LOS, due primarily to a 1.8-day (95% CI -0.95, − 2.61) decrease for the hospitalist group (Fig. 3). Median LOS for patients admitted by general internists or respirologists did not differ by order set use.

### Readmissions

Neither order set implementation, nor order set use in the post-implementation period were associated with changes in readmissions or ED visits for all three physician groups (Tables 3 and 4 and Additional file 1 Table S1). Overall in-hospital mortality did not change with order set implementation, but was lower in the hospitalist group with order set use.

**Table 3:** Readmissions, emergency department visits and in-hospital mortality for pre-post implementation analysis

Results	Pre-implementation (*n* = 556)	Post-implementation (*n* = 857)	*p*-value
7-day readmission, *n* (%)	33 (5.9)	60 (7.0)	0.430
30-day readmission, *n*, (%)	91 (16.4)	166 (19.4)	0.153
90-day readmission, *n*, (%)	170 (30.6)	299 (34.9)	0.093
7-day ED visits, *n* (%)	42 (7.6)	55 (6.4)	0.409
30-day ED visits, *n* (%)	124 (22.3)	196 (22.9)	0.803
In-hospital mortality, *n*, (%)	19 (3.4)	31 (3.6)	0.842

*ED* = emergency department

**Table 4:** Readmissions, emergency department visits and in-hospital mortality for post-implementation analysis

Results	Respirologist			General internist			Hospitalist		
	No order set (*n* = 64)	Order set (*n* = 33)	*p* value	No order set (*n* = 148)	Order set (*n* = 59)	*p* value	No order set (*n* = 239)	Order set (*n* = 314)	*p* value
7-day readmission, *n* (%)	4 (6.3)	2 (6.1)	0.971	18 (12.2)	4 (6.8)	0.257	13 (5.4)	19 (6.1)	0.760
30-day readmission, *n* (%)	19 (29.7)	5 (15.2)	0.116	29 (19.6)	14 (23.7)	0.508	41 (17.2)	58 (18.5)	0.689
90-day readmission, *n* (%)	30 (46.9)	13 (39.4)	0.482	59 (39.9)	19 (32.2)	0.305	75 (31.4)	103 (32.8)	0.723
7-day ED visits, *n* (%)	2 (3.1)	1 (3.0)	0.980	12 (8.1)	3 (5.1)	0.449	15 (6.3)	22 (7.0)	0.734
30-day ED visits, *n* (%)	18 (28.1)	5 (15.2)	0.155	28 (18.9)	14 (23.7)	0.437	54 (22.6)	77 (24.5)	0.597
In-hospital mortality, *n* (%)	0	1 (3.0)	0.162	8 (5.4)	5 (8.5)	0.411	12 (5)	5 (1.6)	0.021

*ED* = emergency department

## Discussion

This is the largest study to evaluate the impact of a standardized, guideline-based electronic order set for AECOPD on hospital LOS. We performed two analyses: a comparison of LOS before and after the order set was implemented, and a comparison of patients admitted with and without the order set after implementation. The results revealed that order set implementation did not result in an overall LOS reduction, perhaps because only 47% of admitting physicians used it. However, the post-implementation analysis revealed that when it was used, the order set was associated with a LOS reduction of 1.15 days. Use of the order set by hospitalists, who admitted 65% of AECOPD patients in the post-implementation cohort, resulted in the largest LOS reduction of 1.8 days. Importantly, there was no increase in either ED or hospital readmissions, suggesting that earlier discharge resulting from order set use did not occur at the expense of harm to the patient.

Our findings extend observations from two recent studies examining the impact of order sets on AECOPD care. In a pre-post design of 243 patients hospitalized with AECOPD, Kitchlu et al. showed that implementation of an order set improved the quality of admission orders using pre-specified measures of guidelines-based care. [16] Using a similar design in a study of 275 patients, Brown et al. showed that physician prescribing practices for AECOPD could be improved with an electronic order set. [13] Secondary analyses in both studies revealed LOS reductions without an increase in readmissions. The current study extends this work by demonstrating an improvement in hospital LOS in a larger study cohort. Unlike the previous studies, which reported on pre-post effects of order set implementation, we specifically examined the effect of actual use of the order set, demonstrating that it could reduce LOS compared to patients admitted without the order set. Furthermore, the stepped wedge design is robust to secular trends in care delivery and LOS of AECOPD patients, and allowed for subgroup analyses by different admitting physician groups. Both our study and the previous studies provide a strong rationale for the standardization of inpatient AECOPD care using computerized order sets.

A standardized order set is appealing due to high variability in inpatient AECOPD management. [9] Previous studies of order sets for AECOPD demonstrated more consistent use of systemic corticosteroids, appropriate antibiotics, and allied health providers such as physiotherapists, [13, 16] all of which have been shown to reduce hospital LOS. [27–29] The current order set similarly prompted admitting physicians to use these therapies, and pre-selection of some items (e.g., bronchodilator delivery, physiotherapy referral) provided additional clinical decision support around guidelines-based care.

The LOS reduction observed after order set implementation was driven by improvements for patients admitted by hospitalists, with no differences observed in the other physician groups; this was an interesting finding with many possible explanations. First, Sandhu et al. demonstrated high variability in AECOPD management among all specialties, with deviation from clinical guidelines occurring more often when care was provided by physicians other than respirologists. [9] This variability may be due to the diversity of medical problems managed by hospitalists, which could lead to a less uniform approach to inpatient AECOPD management. Thus, it is possible that the opportunity for standardization using an order set was greater for hospitalists than for other specialties. Second, heart failure and diabetes were more prevalent in patients admitted under general internists compared to hospitalist patients. These conditions have both been associated with longer LOS in patients with AECOPD, [30] and are unlikely to be impacted by order set use. Third, patients presenting with respiratory failure requiring noninvasive ventilation were only admitted by general internists or respirologists; these indicators of more severe AECOPD were not systematically captured, but could have reduced the effectiveness of the order set. [31] Although the order set’s impact seemed to be isolated to only one admitting group, the 1.8-day reduction in median LOS is an important finding since hospitalists were responsible for providing almost two thirds of inpatient AECOPD care in our study, and this is likely to be similar in other large, tertiary-care urban hospitals in North America.

The order set was used by 47% of admitting physicians during the study period. This low uptake is a consistent finding for voluntary order sets [13, 16, 32, 33] and is a known limitation of their use. [34] Respirologists and general internists used the order set less frequently than hospitalists, for a number of possible reasons. First, the complexity of the patient’s presentation (e.g., respiratory failure requiring noninvasive ventilation) may have made the order set less applicable at the time of admission. Second, respirologists may have greater perceived self-efficacy with AECOPD management, leading them to admit AECOPD patients without using an order set. Finally, whereas the AECOPD order set was the only respiratory order set embedded in the CPOE system, several admission order sets for medical problems typically admitted under hospitalists (e.g., pneumonia, heart failure) were already embedded. Thus, hospitalists may have more experience in order set use compared to other physicians. Importantly, end users were consulted to ensure the order set was intuitive and minimally disruptive to clinical workflow; these factors have been shown to increase uptake of clinical decision support systems such as standardized order sets. [14, 17, 18] The increase in order set use over time suggested that admitting physicians found it useful.

This study has a number of limitations. First, the non-randomized study design raises the possibility that improvements in LOS were due to other differences between groups admitted with and without the order set. However, when implementing complex healthcare interventions such as the AECOPD order set, traditional randomized controlled trials are impractical due to logistical constraints and the risk of contamination within clinical provider groups. The methodologically robust stepped wedge design minimized contamination by allowing implementation and evaluation of the order set in clusters [35] while still analyzing the effect of order sets within physician groups.

Second, the use of administrative data prevented analysis of patient characteristics that might have influenced a physician’s decision to use the order set; it is thus possible that the reduced LOS in the post-implementation period is due to order set use in less complex cases. While our findings are consistent with studies performed in different geographic and clinical settings, [13, 16] we acknowledge the importance of future studies to examine whether COPD severity, presentation acuity, or use of specific interventions (e.g., noninvasive ventilation) impact order set use. Such an analysis would help to tailor strategies aimed at increasing order set uptake by specific physician groups.

The lack of clinical data on COPD severity (e.g., spirometry) or baseline performance status also precluded the determination of differential effects of the order set between COPD subgroups; it is also possible that these factors influenced LOS or readmission rates independently of the order set. However, this information is also often not available to clinicians at the time of admission. Thus, we chose to develop an order set that could be used for all patients admitted with AECOPD, consistent with actual clinical practice. Our statistical models did account for age, sex and clinically relevant comorbidities, indicating that our results were robust to these covariates. Future studies could further evaluate how patient characteristics impact order set use and outcomes from the order set.

Finally, we did not analyze individual components of the AECOPD order set, and thus do not know which orders were actually selected or executed (e.g., physiotherapy referral). We also cannot confirm whether there was concordance between pre-checked orders and actual orders selected by admitting physicians. These components may have differential impact on LOS and could help refine the order set. An understanding of how individual components were used may also help to identify areas for focused quality improvement. While the intent of this study was to evaluate the effectiveness of a comprehensive bundle of orders, the improvement in LOS provides compelling evidence to justify a secondary analysis of individual order set components.

## Conclusion

In conclusion, this study found that when a standardized electronic order set was used to admit patients with AECOPD, LOS was reduced without increasing readmissions. Innovations such as order sets have the potential to lessen the burden of AECOPD hospitalizations on both patients and the healthcare system, and justify additional studies of clinical decision support tools for AECOPD.

## Additional files

Additional file 1: Supplement with additional details on methods and results (DOCX 28 kb)

Additional file 2: Screenshot of AECOPD order set (PDF 400 kb)

Additional file 3: **Figure S1.** Monthly percentage of patients admitted using AECOPD order set during study period. Vertical lines represent implementation start dates for each physician specialty. Respirologist represented by hatched line; general internist represented by black solid line; hospitalist represented by grey solid line. Reported probabilities are for linear trends from time series models for each physician specialty. These models showed no evidence of seasonality or auto-regression (JPG 112 kb)

## Abbreviations

AECOPD: Acute exacerbation of chronic obstructive pulmonary disease
CI: Confidence interval
COPD: Chronic obstructive pulmonary disease
CPOE: Computerized physician order entry
ED: Emergency department
LOS: Length of stay
