# Supplementary material for: Effectiveness of a standardized electronic admission order set for acute exacerbation of chronic obstructive pulmonary disease
Source: BMC Pulm Med. 2018 May 30;18:93. doi: 10.1186/s12890-018-0657-x (PMC5975274; doi:10.1186/s12890-018-0657-x)
Supplement: Supplementary file 1 — Supplement with additional details on methods and results (DOCX 28 kb) [file 12890_2018_657_MOESM1_ESM.docx]

**ADDITIONAL FILE 1**

Effectiveness of a Standardized Electronic Admission Order Set for Acute Exacerbation of Chronic Obstructive Pulmonary Disease

Sachin R. Pendharkar, Maria B. Ospina, Danielle A. Southern, Naushad Hirani, Jim Graham, Peter Faris, Mohit Bhutani, Richard Leigh, Christopher H. Mody, Michael K. Stickland

# Methods

## Order Set Development

The order set was based on Canadian Thoracic Society guidelines for the management of chronic obstructive pulmonary disease (COPD). [1] It contained recommended testing, medication and dosing, consultations, and discharge planning interventions specific to patients with acute exacerbation of COPD (AECOPD). It was built into the hospital’s existing computerized physician order entry (CPOE) system, Sunrise Clinical Manager (Allscripts Solutions, Chicago IL), both as a standalone order set and as part of pre-existing medical admission order sets. Screenshots of the order set are shown in Additional file 2.

Medications were grouped into categories (rescue bronchodilators, controller medications, antibiotics, corticosteroids) and options within each category were listed in order by preferred mode of delivery (e.g. oral before parenteral). Antibiotic choices were also categorized based on whether the AECOPD was simple or complicated, as defined in the Canadian Thoracic Society guidelines. [1]

In areas where experience indicated high variation from established guidelines (e.g., mode of bronchodilator delivery), interventions were pre-selected. These pre-selected interventions could be changed at the admitting clinician’s discretion. Pre-selection of medications was also expected to improve clinical workflow since the admitting physician would not have to individually select these medications. The working group chose not to pre-select antibiotic medication and dose, as the frequency with which a pre-selected antibiotic would need to be changed was felt to be high enough that clinical workflow would be interrupted. The order set was reviewed with frontline physicians to ensure ease of use.

## Implementation

The order set was implemented using a stepped wedge design [2] among three physician groups who admit patients with AECOPD: respirologists, general internists, and hospitalists (physicians who are certified by the College of Family Physicians whose primary activity is the admission and care of hospitalized patients). The intervention was implemented sequentially within clusters (physician groups) with each cluster acting as its own control. Study outcome data were collected at baseline and at each implementation ‘step’. This design was selected to permit robust evaluation of the order set, including controlling for secular trends in length of stay, despite the need to implement it sequentially due to logistical constraints.

Implementation among respirologists, general internists and hospitalists occurred in March, May and August 2013, respectively. Respirologists and general internists underwent implementation sequentially to minimize the risk of contamination from clinical trainees rotating through both specialties. Patients admitted to a clinical service before order set implementation in the corresponding physician group were considered historical controls even though the order set was available to all physicians throughout the study.

Prior to each planned implementation step, the order set project team met with members of the physician and allied health groups to introduce the order set. Trainees were also informed of the order set by electronic mail at the beginning of each clinical training rotation. Utilization of the order set by each individual physician was voluntary. Although the order set was available to any physician throughout the pilot, it was only integrated into specialty-specific order sets when introduced to that specialty group. Monthly statistics on order set use were posted in clinical areas, and the project team was available to physicians for any questions related to its use.

## Analysis

Patient demographic, comorbidity and hospitalization data were obtained from the hospital’s Discharge Abstract Database and order set usage data were obtained from the CPOE system. Deterministic data linkage across databases was based on the patient’s provincial health number. [3] Patients were excluded from the analysis if they were transferred to another acute care or long term care facility or if hospital length of stay exceeded 90 days.

The primary outcome was hospital length of stay for patients admitted during the order set implementation period compared to those admitted during the previous 12 months (pre-post implementation analysis). Secondary outcomes included: hospital length of stay of patients admitted with and without the order set after order set implementation (post-implementation analysis); all-cause readmissions at 7, 30 and 90 days after discharge; ED visits at 7 and 30 days; and in-hospital mortality. The unit of analysis was the hospital admission; thus, patients with multiple admissions may have been included in order-set and no order-set groups, in both the pre-post implementation and post-implementation analysis.

Historic administrative data were used to determine the anticipated volumes of patients treated by each physician group, and distribution of their length of stay. Calculations accounted for study design, and determined that with an alpha level of 0.05, 460 admissions were required to achieve 80% power to detect a 1 day (17%) decrease in the median length of stay.

Descriptive analyses were conducted using proportions and percentages for categorical data, and means with standard deviations (SD) or median and interquartile range for continuous data. Univariate analysis of continuous data was conducted using two-sample t tests or Mann-Whitney test (for non-normally distributed data). Order set uptake was evaluated using time series analysis for each physician specialty group.

Median regression models were constructed to assess the impact of the order set on length of stay. [4-6] Median regression was selected over other modelling methods because highly skewed length of stay data would require transformation before they could be analyzed using other techniques. Such transformation would lead to results that were not easily interpreted in a clinical setting; in contrast, median regression permitted us to analyze the data without transformation. The models included both a term for the intervention (introduction of the order set for the pre-post implementation analysis and no order set vs order set for the post-implementation analysis) and a fixed term for each of the three physician groups. Both unadjusted and adjusted models were constructed to account for demographic and clinical factors that could affect hospital length of stay; confounders included age, sex, and five clinically relevant comorbidities selected from the Charlson Comorbidity Index [7] (heart failure, dementia, mild or severe liver disease, renal disease, and diabetes). These selected comorbidities accounted for almost all Charlson comorbidities in the study cohort (Somers’ D = 0.94; 95% confidence interval [CI] 0.93, 0.96). For all models, we first tested an intervention by step interaction, and then removed the interaction term to obtain an overall estimate of effect. This analysis revealed no evidence that the effect of the order set implementation depended on the admitting physician group. For the post-implementation analysis, the effect of order set use was dependent on admitting physician group; this interaction was not significant when comorbidity covariates were considered in the model

As each step can be considered a replication of the intervention, forest plots were used to illustrate both the effects within each step as well as the overall estimate of effects. Both adjusted and unadjusted results are presented within each forest plot. A sensitivity analysis was performed after removing all patients who died within 90 days with no significant difference observed. Logistic regression was used to adjust 30-day readmission odds ratios (OR) for age, sex, comorbidity and admitting physician specialty.

All analyses were performed using SAS version 9.3 (Cary, NC) or R version 3.2.3; [8] p <0.05 was considered statistically significant.

# REFERENCES

1. O'Donnell DE, Hernandez P, Kaplan A, Aaron S, Bourbeau J, Marciniuk D, Balter M, Ford G, Gervais A, Lacasse Y, Maltais F, Road J, Rocker G, Sin D, Sinuff T, Voduc N. Canadian Thoracic Society recommendations for management of chronic obstructive pulmonary disease - 2008 update - highlights for primary care. Can Respir J 2008;15 Suppl A:1A-8A.
2. Hemming K, Haines TP, Chilton PJ, Girling AJ, Lilford RJ. The stepped wedge cluster randomized trial: rationale, design, analysis, and reporting. Br Med J 2015;350:h391.
3. Howe GR. Use of computerized record linkage in cohort studies. Epidemiol Rev 1998;20:112-21.
4. Koenker R. Quantile Regression [computer program]. https://CRAN.R-project.org/package=quantreg. 2015.
5. Koenker R, Bassett Jr G. Regression quantiles. Econometrica: J Econom Soc 1978:33-50.
6. Portnoy S, Koenker R. The gaussian hare and the laplacian tortoise: Computability of squared-error versus absolute-error estimators. Stat Sci 1997;12(4):279-300.
7. Charlson ME, Pompei P, Ales KL, Mackenzie CR. A new method of classifying prognostic comorbidity in longitudinal studies: development and validation. J Chronic Dis 1987;40:373-83.
8. R: A language and environment for statistical computing [computer program]. Vienna, Austria: R Foundation for Statistical Computing; 2015.

.

**TABLES**

Table E1: Regression Analysis for Readmissions

|  | **Post vs. Pre-Implementation** | **Order Set vs. No Order Set** |
| --- | --- | --- |
| **30-day Readmission** |  |  |
| **Crude OR (95% CI)** | 1.29 (0.93,1.63) | 1.08 (0.8,1.45) |
| **Adjusted OR (95% CI)** | 1.16 (0.87,1.55) | 1.17 (0.87,1.59) |
| **30-day ED visit** |  |  |
| **Crude OR (95% CI)** | 1.03 (0.8,1.33) | 1.08 (0.8,1.42) |
| **Adjusted OR (95% CI)** | 1.03 (0.8,1.34) | 1.07 (0.82,1.41) |

OR = odds ratio; CI = confidence interval
