# Supplementary material for: Effectiveness of a standardized electronic admission order set for acute exacerbation of chronic obstructive pulmonary disease
Source: BMC Pulm Med. 2018 May 30;18:93. doi: 10.1186/s12890-018-0657-x (PMC5975274; doi:10.1186/s12890-018-0657-x)
Supplement: Supplementary file 2 — Screenshot of AECOPD order set (PDF 400 kb) [file 12890_2018_657_MOESM2_ESM.pdf]

Additional file 2: Screen shot of order set

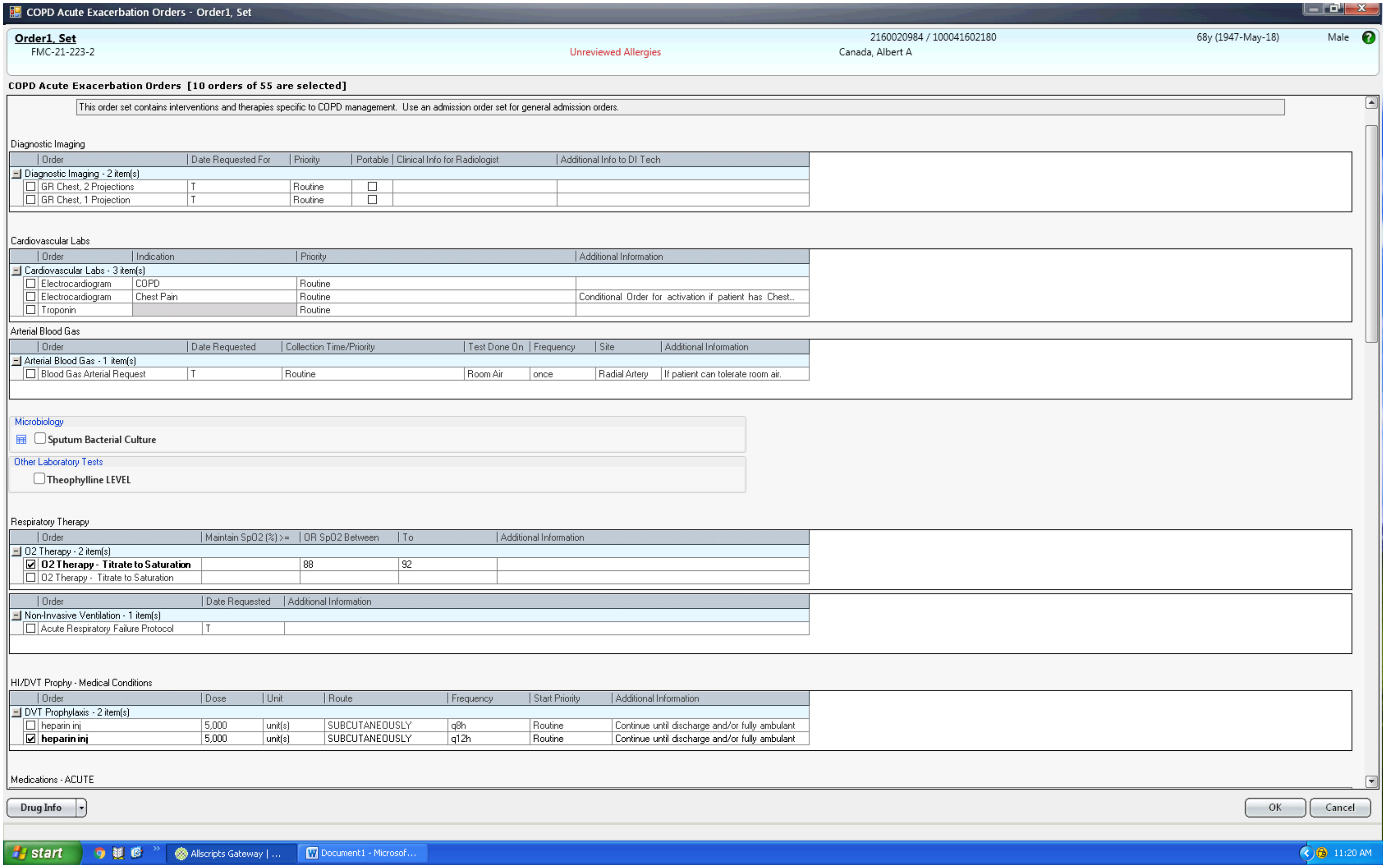

COPD Acute Exacerbation Orders - Order1, Set

Order1, Set
FMC-21-223-2

Unreviewed Allergies

2160020984 / 100041602180
Canada, Albert A

68y (1947-May-18)

Male

COPD Acute Exacerbation Orders [10 orders of 55 are selected]

This order set contains interventions and therapies specific to COPD management. Use an admission order set for general admission orders.

Diagnostic Imaging

Order	Date Requested For	Priority	Portable	Clinical Info for Radiologist	Additional Info to DI Tech
Diagnostic Imaging - 2 item(s)					
☐ GR Chest, 2 Projections	T	Routine	☐		
☐ GR Chest, 1 Projection	T	Routine	☐		

Cardiovascular Labs

Order	Indication	Priority	Additional Information
Cardiovascular Labs - 3 item(s)			
☐ Electrocardiogram	COPD	Routine	
☐ Electrocardiogram	Chest Pain	Routine	Conditional Order for activation if patient has Chest...
☐ Troponin		Routine	

Arterial Blood Gas

Order	Date Requested	Collection Time/Priority	Test Done On	Frequency	Site	Additional Information
Arterial Blood Gas - 1 item(s)						
☐ Blood Gas Arterial Request	T	Routine	Room Air	once	Radial Artery	If patient can tolerate room air.

Microbiology
☐ Sputum Bacterial Culture

Other Laboratory Tests
☐ Theophylline LEVEL

Respiratory Therapy

Order	Maintain SpO2 (%) >=	OR SpO2 Between	To	Additional Information
O2 Therapy - 2 item(s)				
☑ **O2 Therapy - Titrate to Saturation**		88	92	
☐ O2 Therapy - Titrate to Saturation				

Order	Date Requested	Additional Information
Non-Invasive Ventilation - 1 item(s)		
☐ Acute Respiratory Failure Protocol	T	

HI/DVT Prophy - Medical Conditions

Order	Dose	Unit	Route	Frequency	Start Priority	Additional Information
DVT Prophylaxis - 2 item(s)						
☐ heparin inj	5,000	unit(s)	SUBCUTANEOUSLY	q8h	Routine	Continue until discharge and/or fully ambulant
☑ **heparin inj**	5,000	unit(s)	SUBCUTANEOUSLY	q12h	Routine	Continue until discharge and/or fully ambulant

Medications - ACUTE

Drug Info | OK | Cancel

start | Allscripts Gateway | ... | Document1 - Microsof... | 11:20 AM

Additional file 2: Screen shot of order set

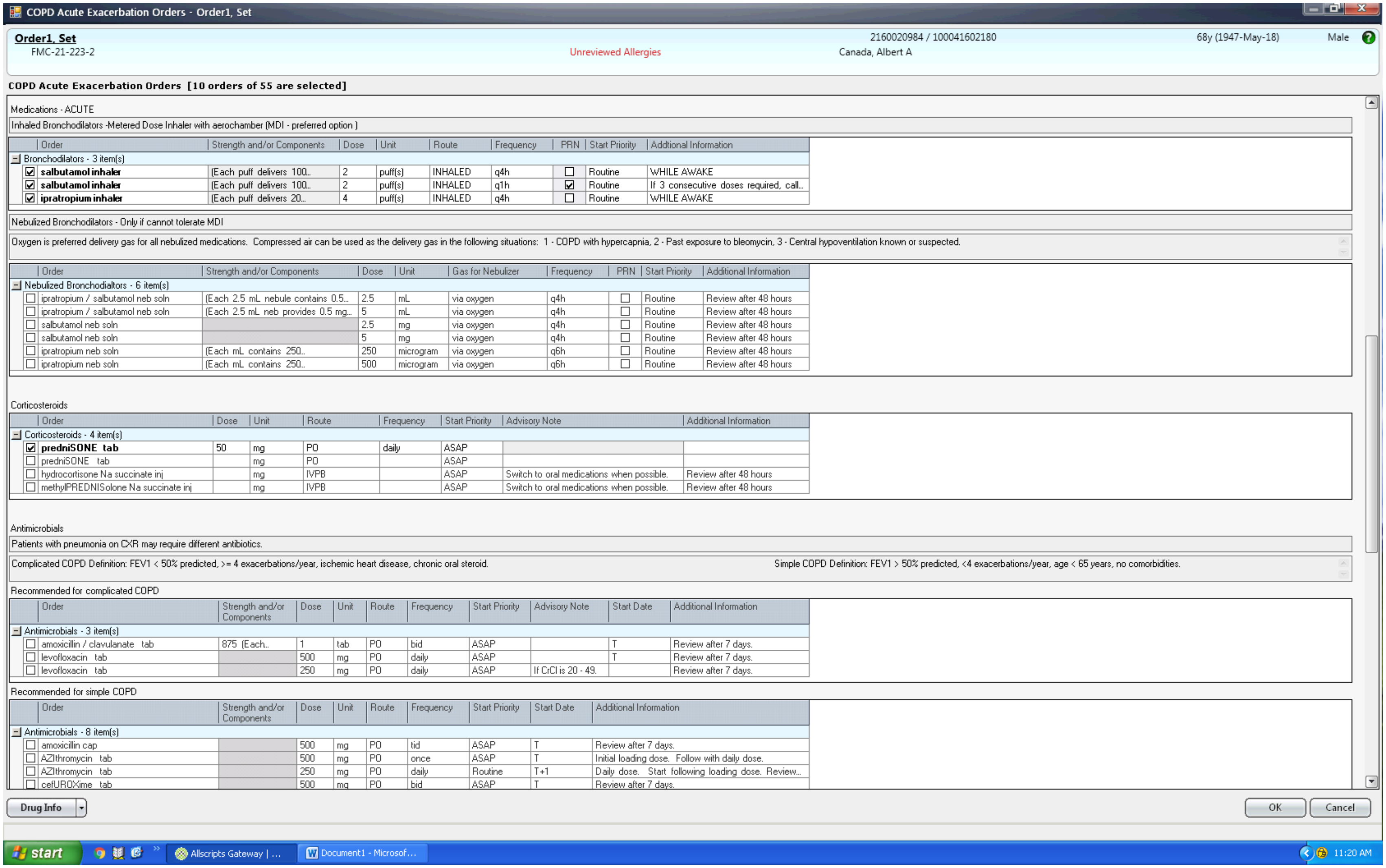

COPD Acute Exacerbation Orders - Order1, Set

Order1, Set
FMC-21-223-2

Unreviewed Allergies

2160020984 / 100041602180
Canada, Albert A

68y (1947-May-18)   Male

COPD Acute Exacerbation Orders [10 orders of 55 are selected]

Medications - ACUTE

Inhaled Bronchodilators -Metered Dose Inhaler with aerochamber (MDI - preferred option)

	Order	Strength and/or Components	Dose	Unit	Route	Frequency	PRN	Start Priority	Addtional Information
	Bronchodilators - 3 item(s)								
☑	**salbutamol inhaler**	(Each puff delivers 100...	2	puff(s)	INHALED	q4h	☐	Routine	WHILE AWAKE
☑	**salbutamol inhaler**	(Each puff delivers 100...	2	puff(s)	INHALED	q1h	☑	Routine	If 3 consecutive doses required, call...
☑	**ipratropium inhaler**	(Each puff delivers 20...	4	puff(s)	INHALED	q4h	☐	Routine	WHILE AWAKE

Nebulized Bronchodilators - Only if cannot tolerate MDI

Oxygen is preferred delivery gas for all nebulized medications. Compressed air can be used as the delivery gas in the following situations: 1 - COPD with hypercapnia, 2 - Past exposure to bleomycin, 3 - Central hypoventilation known or suspected.

	Order	Strength and/or Components	Dose	Unit	Gas for Nebulizer	Frequency	PRN	Start Priority	Additional Information
	Nebulized Bronchodialtors - 6 item(s)								
☐	ipratropium / salbutamol neb soln	(Each 2.5 mL nebule contains 0.5...	2.5	mL	via oxygen	q4h	☐	Routine	Review after 48 hours
☐	ipratropium / salbutamol neb soln	(Each 2.5 mL neb provides 0.5 mg...	5	mL	via oxygen	q4h	☐	Routine	Review after 48 hours
☐	salbutamol neb soln		2.5	mg	via oxygen	q4h	☐	Routine	Review after 48 hours
☐	salbutamol neb soln		5	mg	via oxygen	q4h	☐	Routine	Review after 48 hours
☐	ipratropium neb soln	(Each mL contains 250...	250	microgram	via oxygen	q6h	☐	Routine	Review after 48 hours
☐	ipratropium neb soln	(Each mL contains 250...	500	microgram	via oxygen	q6h	☐	Routine	Review after 48 hours

Corticosteroids

	Order	Dose	Unit	Route	Frequency	Start Priority	Advisory Note	Additional Information
	Corticosteroids - 4 item(s)							
☑	**predniSONE tab**	50	mg	PO	daily	ASAP		
☐	predniSONE tab		mg	PO		ASAP		
☐	hydrocortisone Na succinate inj		mg	IVPB		ASAP	Switch to oral medications when possible.	Review after 48 hours
☐	methylPREDNISolone Na succinate inj		mg	IVPB		ASAP	Switch to oral medications when possible.	Review after 48 hours

Antimicrobials

Patients with pneumonia on CXR may require different antibiotics.

Complicated COPD Definition: FEV1 < 50% predicted, >= 4 exacerbations/year, ischemic heart disease, chronic oral steroid.

Simple COPD Definition: FEV1 > 50% predicted, <4 exacerbations/year, age < 65 years, no comorbidities.

Recommended for complicated COPD

	Order	Strength and/or Components	Dose	Unit	Route	Frequency	Start Priority	Advisory Note	Start Date	Additional Information
	Antimicrobials - 3 item(s)									
☐	amoxicillin / clavulanate tab	875 (Each..	1	tab	PO	bid	ASAP		T	Review after 7 days.
☐	levofloxacin tab		500	mg	PO	daily	ASAP		T	Review after 7 days.
☐	levofloxacin tab		250	mg	PO	daily	ASAP	If CrCl is 20 - 49.		Review after 7 days.

Recommended for simple COPD

	Order	Strength and/or Components	Dose	Unit	Route	Frequency	Start Priority	Start Date	Additional Information
	Antimicrobials - 8 item(s)								
☐	amoxicillin cap		500	mg	PO	tid	ASAP	T	Review after 7 days.
☐	AZIthromycin tab		500	mg	PO	once	ASAP	T	Initial loading dose. Follow with daily dose.
☐	AZIthromycin tab		250	mg	PO	daily	Routine	T+1	Daily dose. Start following loading dose. Review...
☐	cefUROXime tab		500	mg	PO	bid	ASAP	T	Review after 7 days.

Drug Info   OK   Cancel

start   Allscripts Gateway | ...   Document1 - Microsof...   11:20 AM

Additional file 2: Screen shot of order set

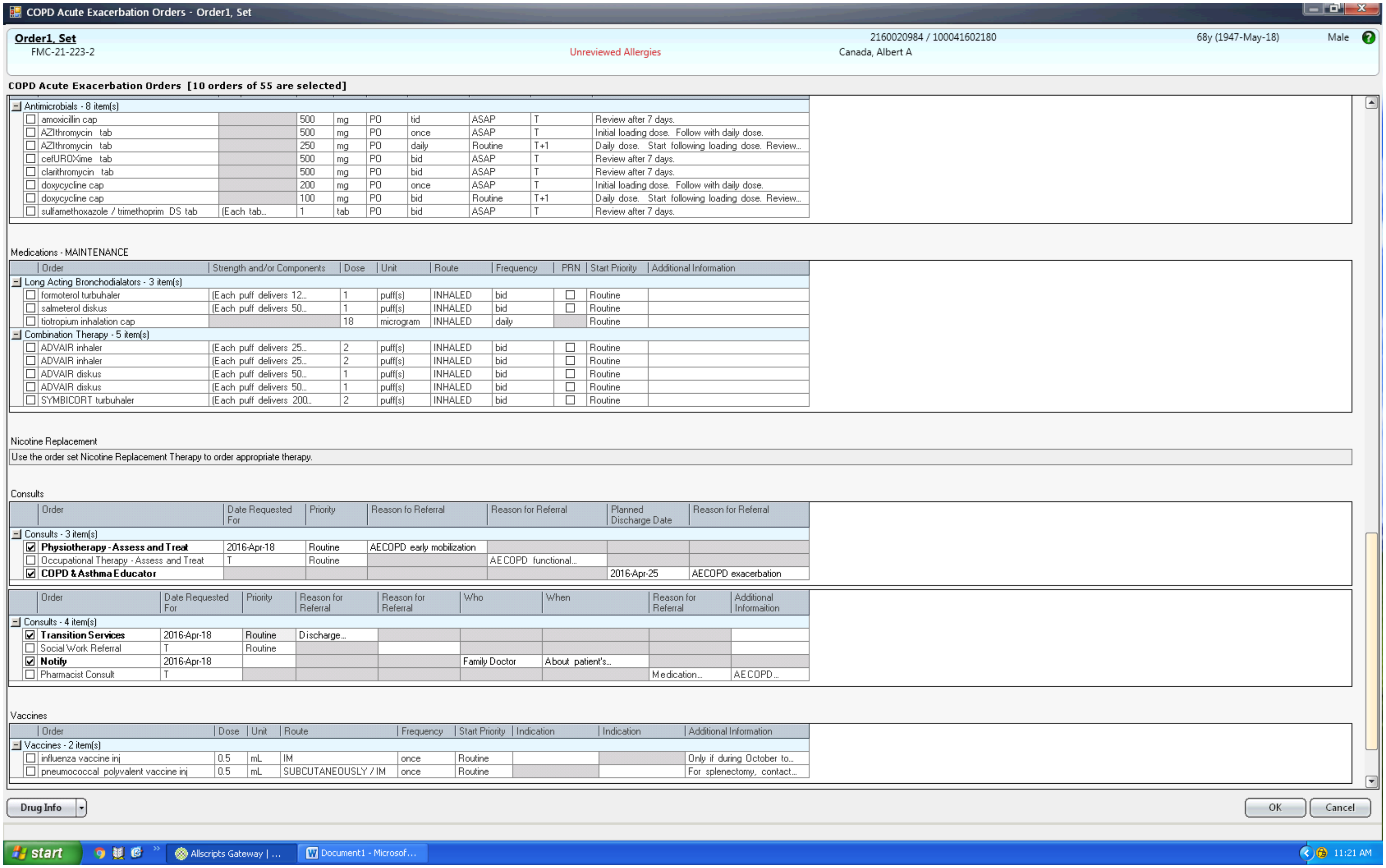

COPD Acute Exacerbation Orders - Order1, Set

Order1, Set
FMC-21-223-2

Unreviewed Allergies

2160020984 / 100041602180
Canada, Albert A

68y (1947-May-18) Male

COPD Acute Exacerbation Orders [10 orders of 55 are selected]

Antimicrobials - 8 item(s)								
☐ amoxicillin cap		500	mg	PO	tid	ASAP	T	Review after 7 days.
☐ AZIthromycin tab		500	mg	PO	once	ASAP	T	Initial loading dose. Follow with daily dose.
☐ AZIthromycin tab		250	mg	PO	daily	Routine	T+1	Daily dose. Start following loading dose. Review...
☐ cefUROXime tab		500	mg	PO	bid	ASAP	T	Review after 7 days.
☐ clarithromycin tab		500	mg	PO	bid	ASAP	T	Review after 7 days.
☐ doxycycline cap		200	mg	PO	once	ASAP	T	Initial loading dose. Follow with daily dose.
☐ doxycycline cap		100	mg	PO	bid	Routine	T+1	Daily dose. Start following loading dose. Review...
☐ sulfamethoxazole / trimethoprim DS tab	(Each tab...	1	tab	PO	bid	ASAP	T	Review after 7 days.

Medications - MAINTENANCE

Order	Strength and/or Components	Dose	Unit	Route	Frequency	PRN	Start Priority	Additional Information
Long Acting Bronchodilators - 3 item(s)								
☐ formoterol turbuhaler	(Each puff delivers 12...	1	puff(s)	INHALED	bid	☐	Routine	
☐ salmeterol diskus	(Each puff delivers 50...	1	puff(s)	INHALED	bid	☐	Routine	
☐ tiotropium inhalation cap		18	microgram	INHALED	daily		Routine	
Combination Therapy - 5 item(s)								
☐ ADVAIR inhaler	(Each puff delivers 25...	2	puff(s)	INHALED	bid	☐	Routine	
☐ ADVAIR inhaler	(Each puff delivers 25...	2	puff(s)	INHALED	bid	☐	Routine	
☐ ADVAIR diskus	(Each puff delivers 50...	1	puff(s)	INHALED	bid	☐	Routine	
☐ ADVAIR diskus	(Each puff delivers 50...	1	puff(s)	INHALED	bid	☐	Routine	
☐ SYMBICORT turbuhaler	(Each puff delivers 200...	2	puff(s)	INHALED	bid	☐	Routine	

Nicotine Replacement

Use the order set Nicotine Replacement Therapy to order appropriate therapy.

Consults

Order	Date Requested For	Priority	Reason fo Referral	Reason for Referral	Planned Discharge Date	Reason for Referral
Consults - 3 item(s)						
☑ **Physiotherapy - Assess and Treat**	2016-Apr-18	Routine	AECOPD early mobilization			
☐ Occupational Therapy - Assess and Treat	T	Routine		AECOPD functional...		
☑ **COPD & Asthma Educator**					2016-Apr-25	AECOPD exacerbation

Order	Date Requested For	Priority	Reason for Referral	Reason for Referral	Who	When	Reason for Referral	Additional Information
Consults - 4 item(s)								
☑ **Transition Services**	2016-Apr-18	Routine	Discharge...					
☐ Social Work Referral	T	Routine						
☑ **Notify**	2016-Apr-18				Family Doctor	About patient's...		
☐ Pharmacist Consult	T						Medication...	AECOPD...

Vaccines

Order	Dose	Unit	Route	Frequency	Start Priority	Indication	Indication	Additional Information
Vaccines - 2 item(s)								
☐ influenza vaccine inj	0.5	mL	IM	once	Routine			Only if during October to...
☐ pneumococcal polyvalent vaccine inj	0.5	mL	SUBCUTANEOUSLY / IM	once	Routine			For splenectomy, contact...

Drug Info OK Cancel

start Allscripts Gateway | ... Document1 - Microsof... 11:21 AM
