# Supplementary figures and images for: Effectiveness of a standardized electronic admission order set for acute exacerbation of chronic obstructive pulmonary disease
Source: BMC Pulm Med. 2018 May 30;18:93. doi: 10.1186/s12890-018-0657-x (PMC5975274; doi:10.1186/s12890-018-0657-x)

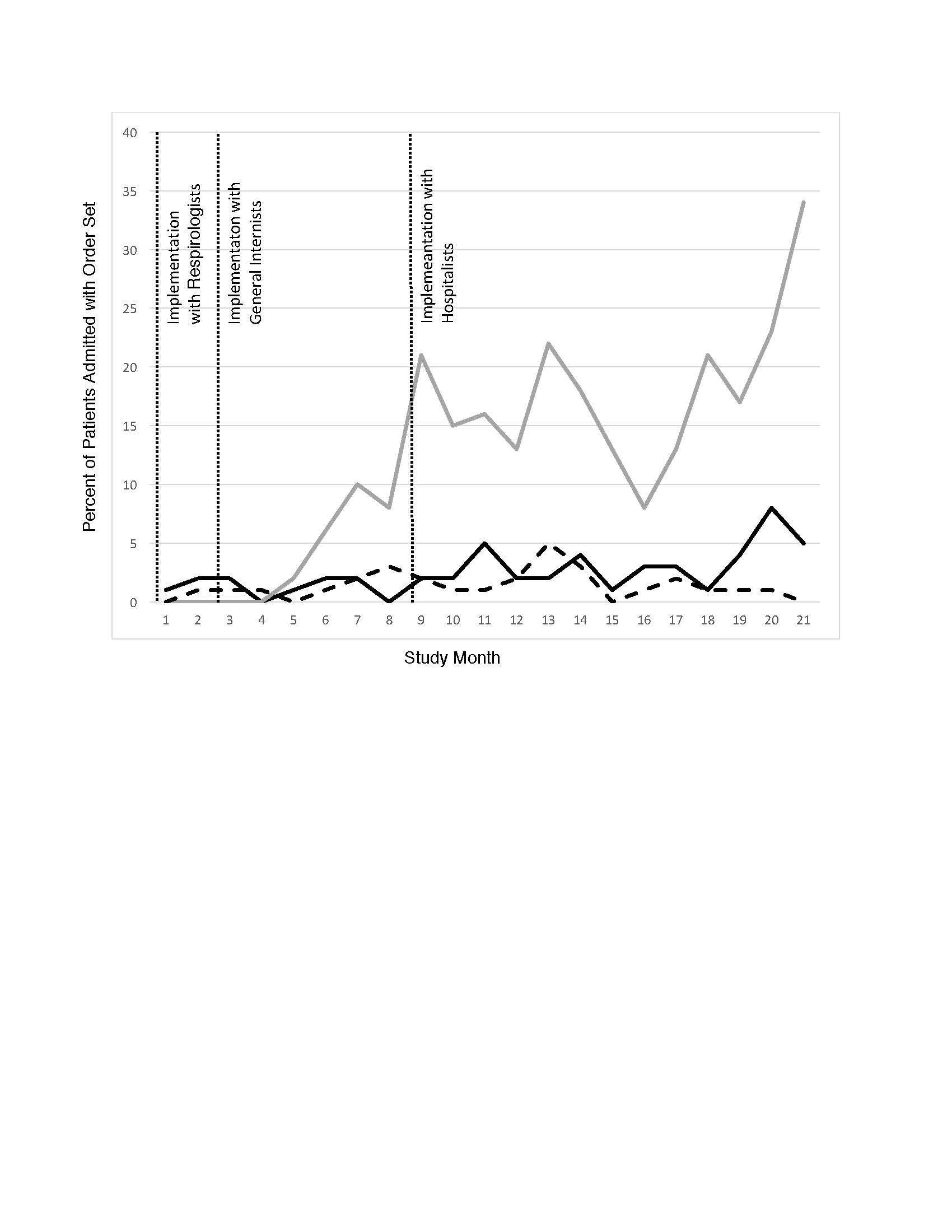

Supplement: Supplementary file 3 — Figure S1. Monthly percentage of patients admitted using AECOPD order set during study period. Vertical lines represent implementation start dates for each physician specialty. Respirologist represented by hatched line; general internist represented by black solid line; hospitalist represented by grey solid line. Reported probabilities are for linear trends from time series models for each physician specialty. These models showed no evidence of seasonality or auto-regression (JPG 112 kb) [file 12890_2018_657_MOESM3_ESM.jpg]
